# Supplementary material for: Mapping species of greatest conservation need and solar energy potential in the arid Southwest for future sustainable development
Source: PeerJ. 2025 Jan 2;13:e18568. doi: 10.7717/peerj.18568 (PMC11700496; doi:10.7717/peerj.18568)

Target Species Habitat Suitability in United States Southwest

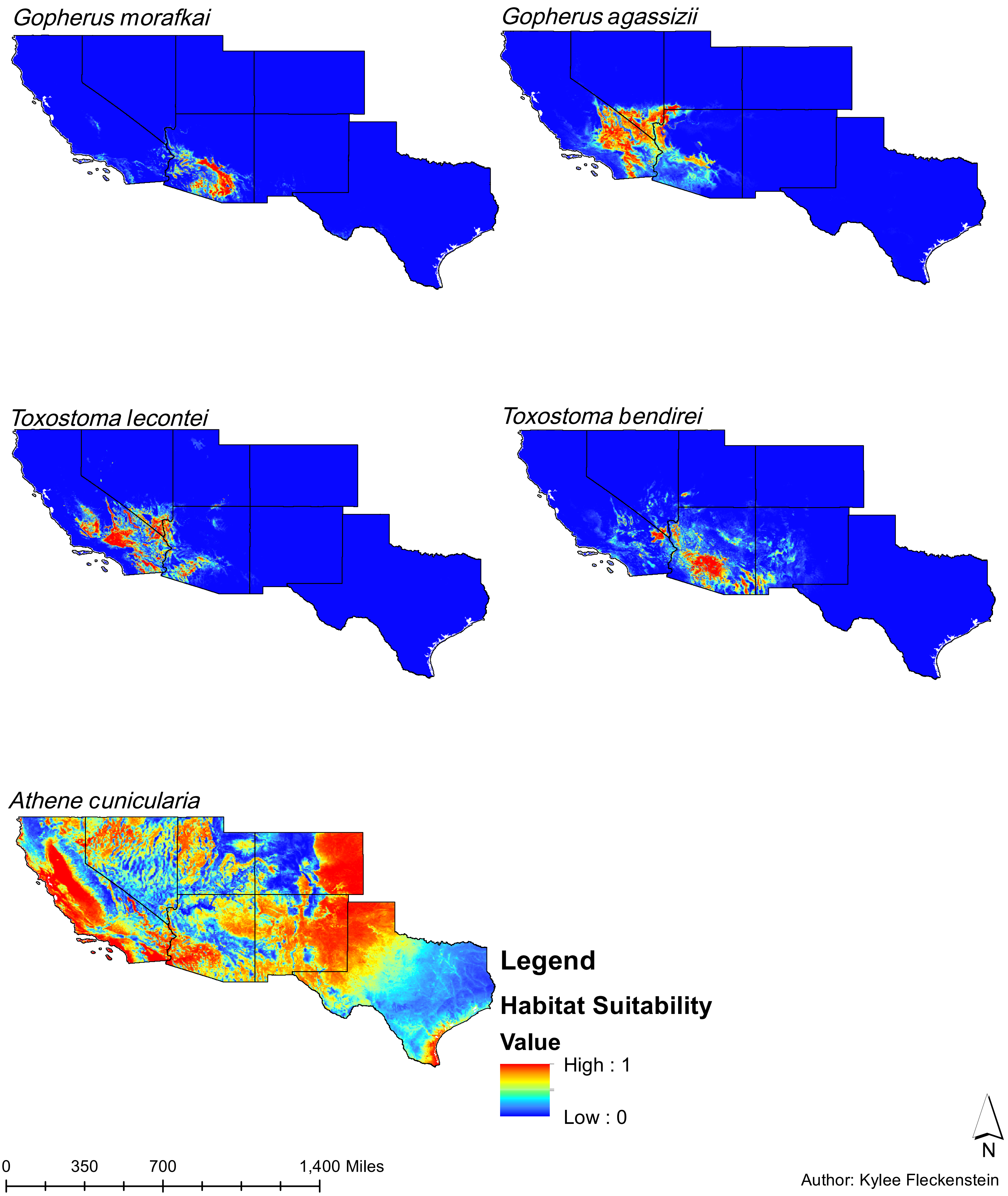

Supplement: Supplemental Information 2 — The habitat suitability of five target species is illustrated —Gopherus morafkai , Gopherus agassizii , Toxostoma bendirei , Athene cunicularia , and Toxostoma lecontei —across the Southwest. Habitat suitability values range from 0 (low suitability) to 1 (high suitability). [file peerj-13-18568-s002.pdf]
