# Supplementary figures and images for: Mapping species of greatest conservation need and solar energy potential in the arid Southwest for future sustainable development
Source: PeerJ. 2025 Jan 2;13:e18568. doi: 10.7717/peerj.18568 (PMC11700496; doi:10.7717/peerj.18568)

# Target Species Predicted Presence in United States Southwest

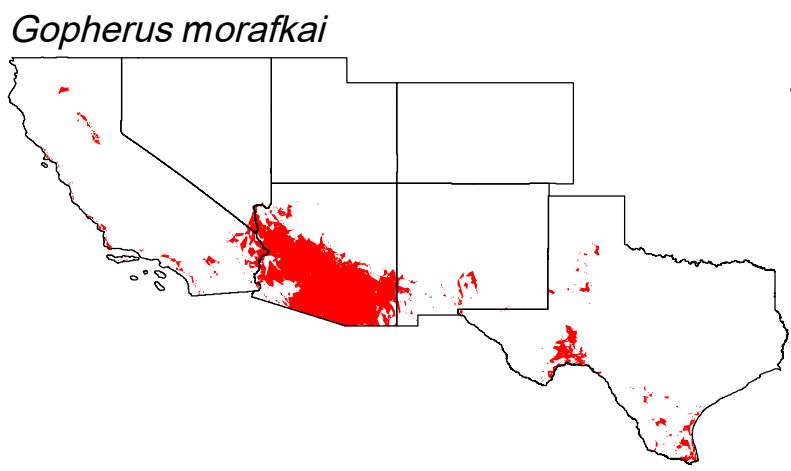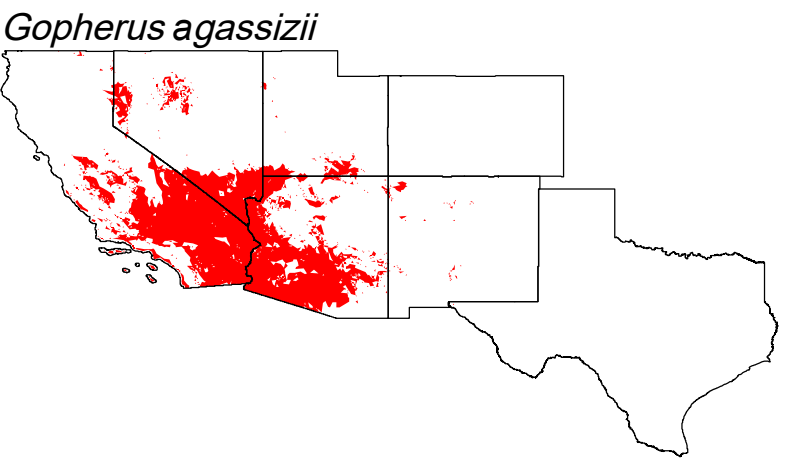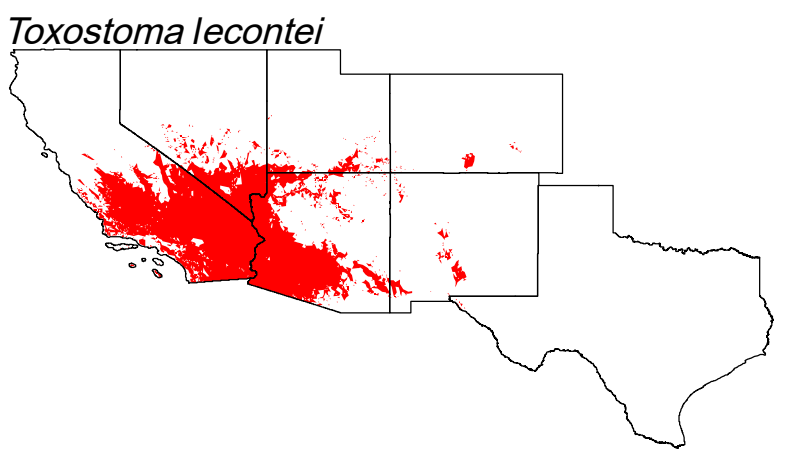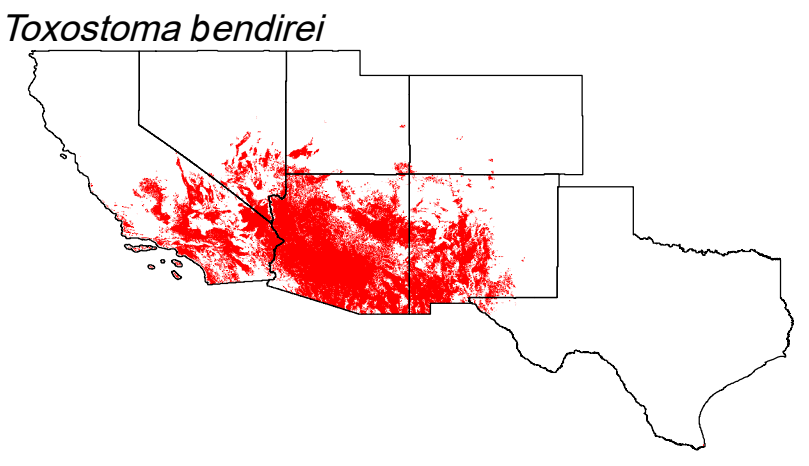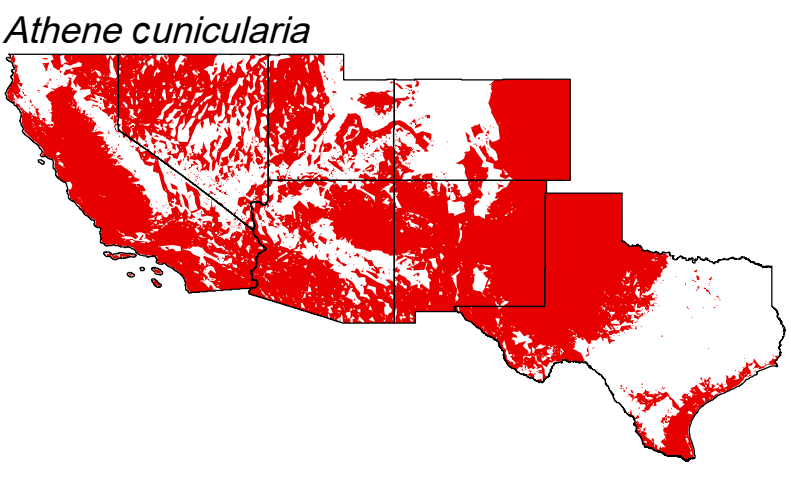

## Legend

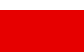 Predicted Presence

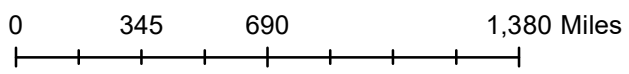

Supplement: Supplemental Information 3 — The predicted presence of the five selected species in the Southwest based on species distribution modeling (SDM). Presence data are aggregated to depict regions with a high likelihood of species occurrence. [file peerj-13-18568-s003.pdf]

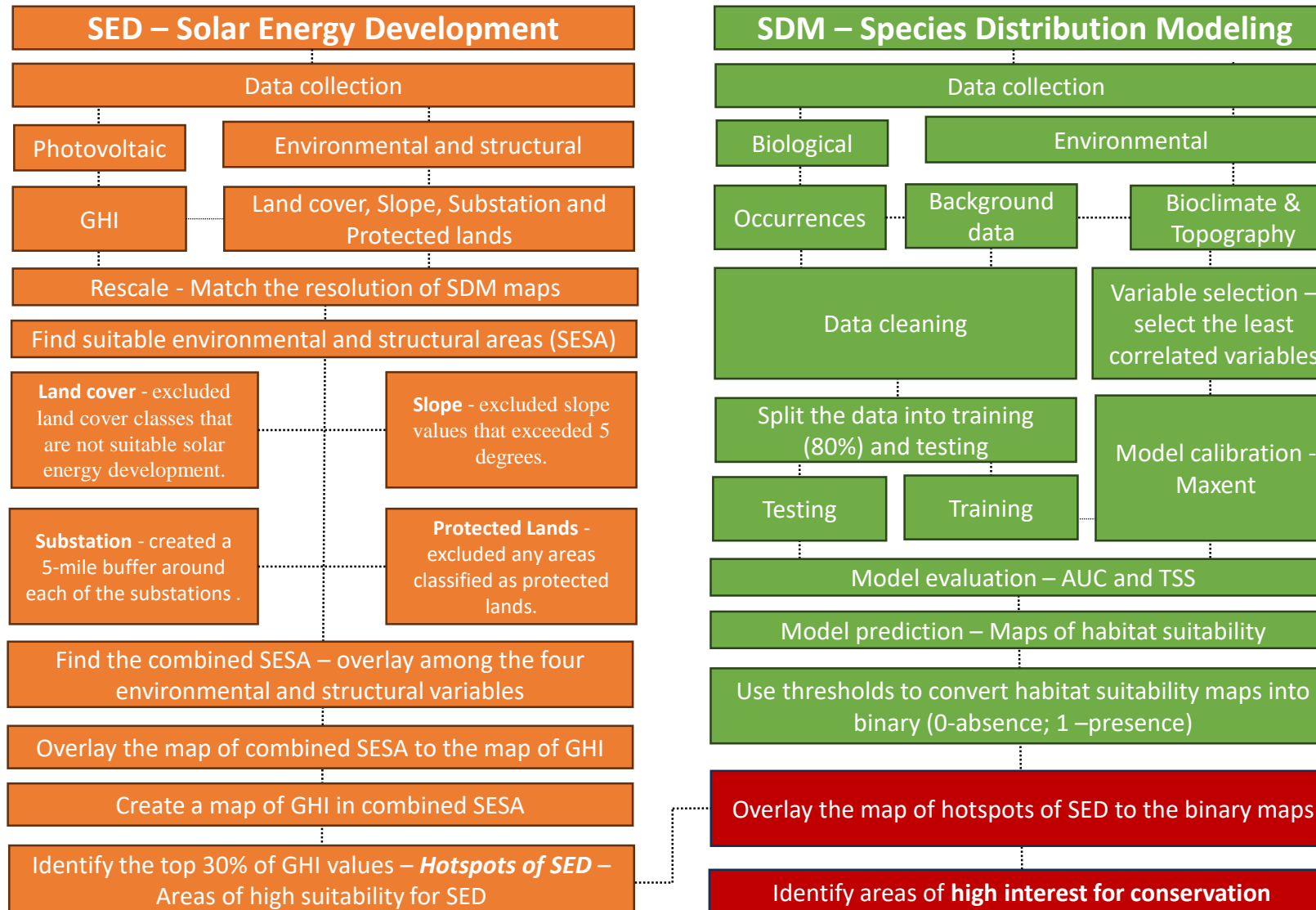

Supplement: Supplemental Information 6 — This flowchart outlines the workflow used in the study to combine site suitability analysis with species distribution modeling. The diagram details each analytical step, including variable selection, habitat suitability modeling, and overlap analysis, providing a comprehensive view of the methodology and decision making. [file peerj-13-18568-s006.pdf]

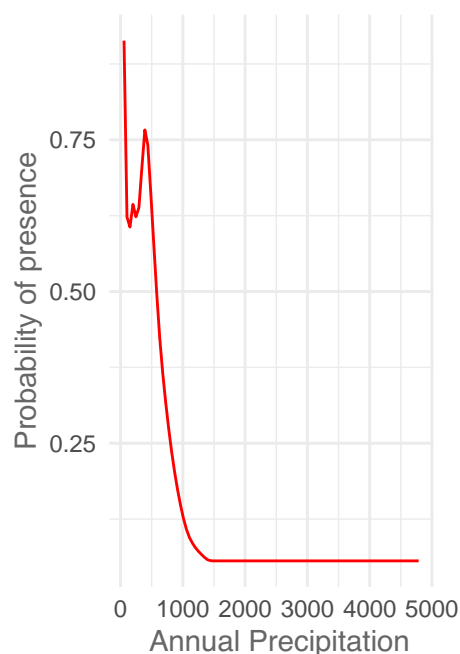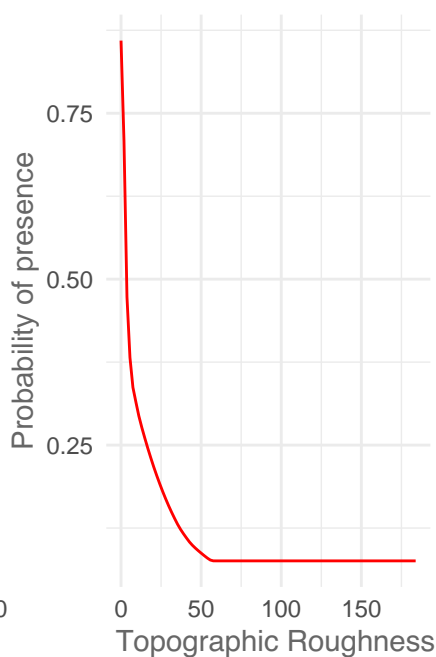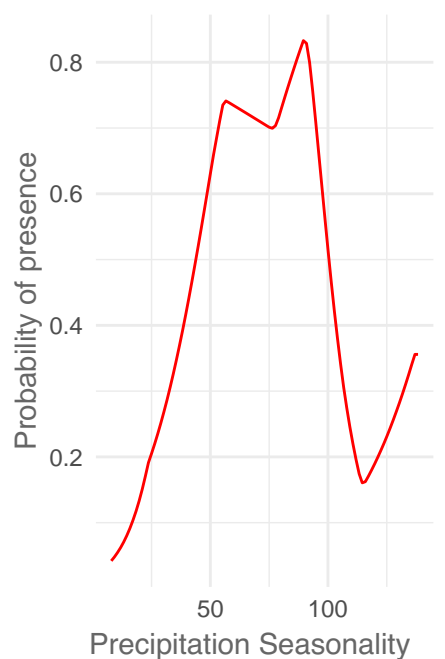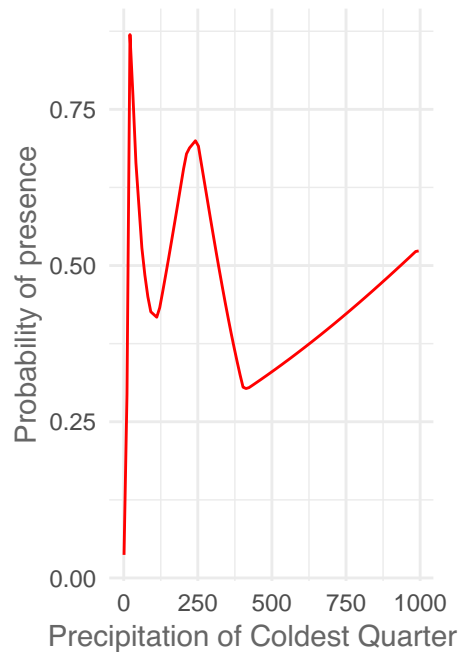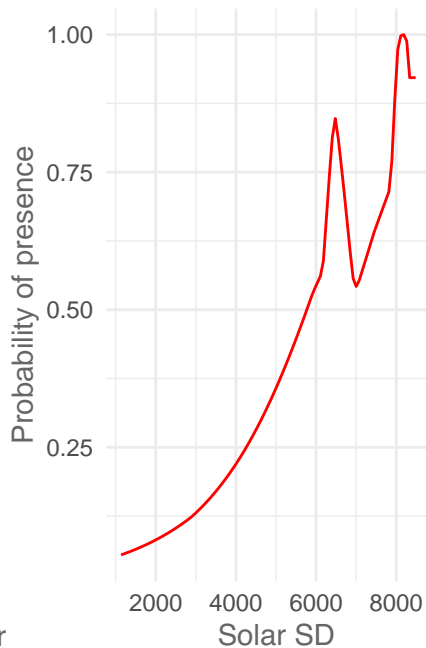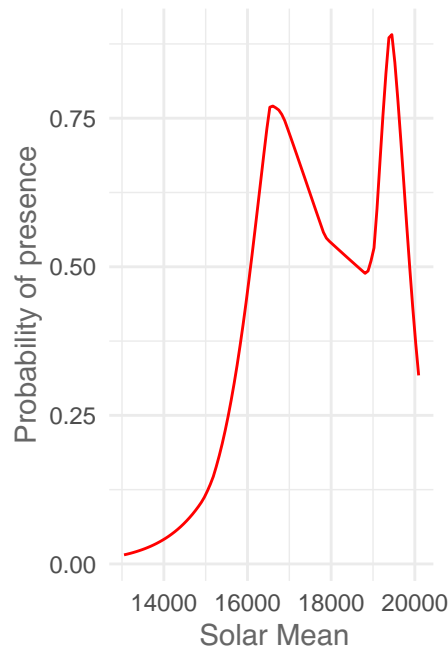

Supplement: Supplemental Information 7 — The variable response curves of key environmental variables influencing the habitat suitability for Athene cunicularia. Each curve represents the relationship between environmental such as precipitation, solar radiation. [file peerj-13-18568-s007.pdf]

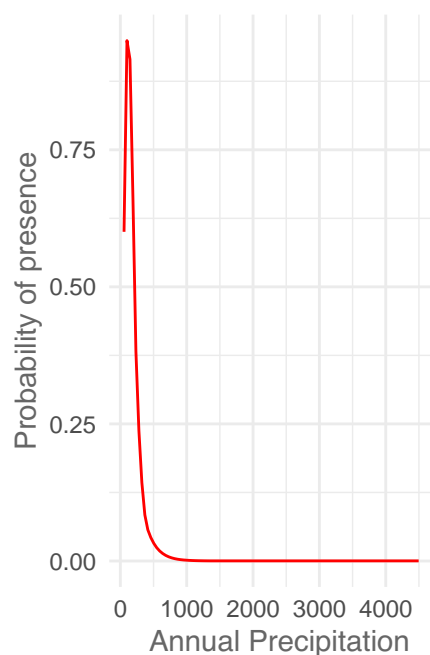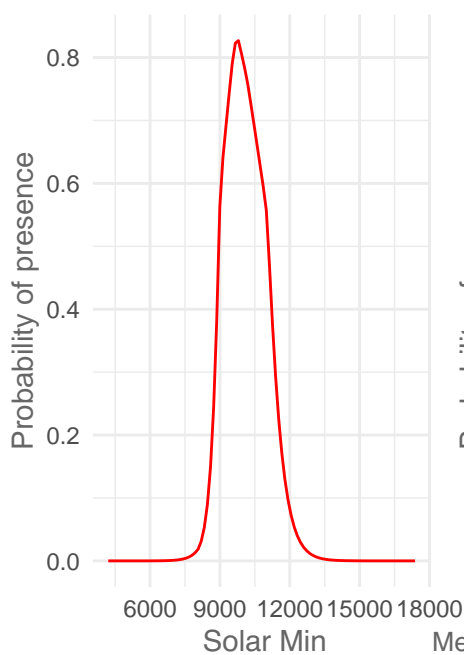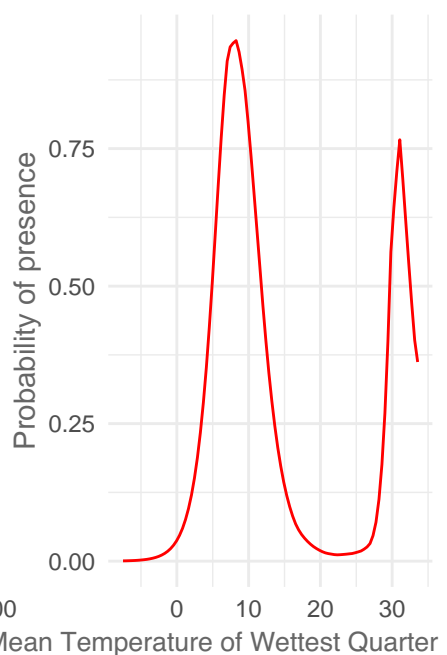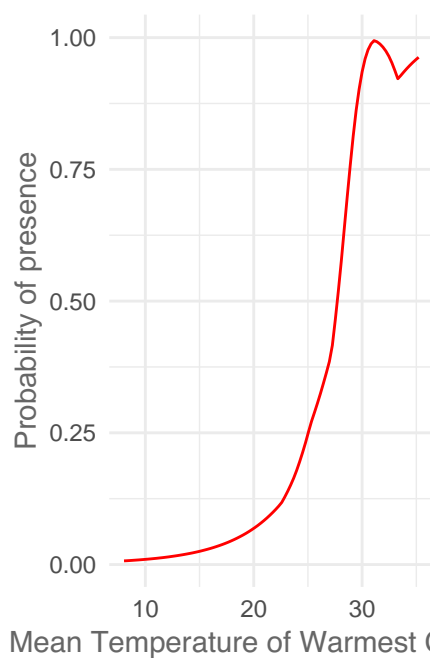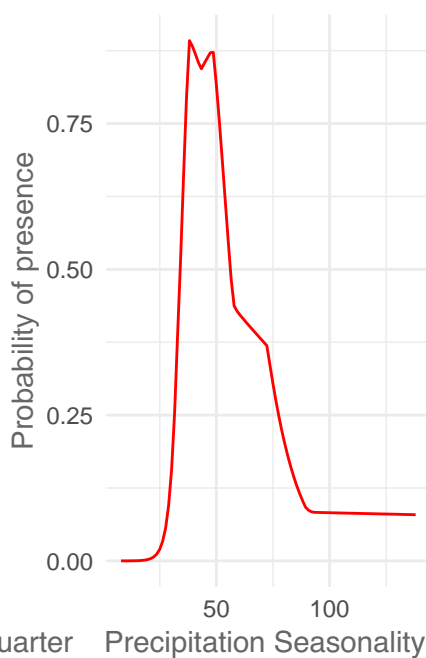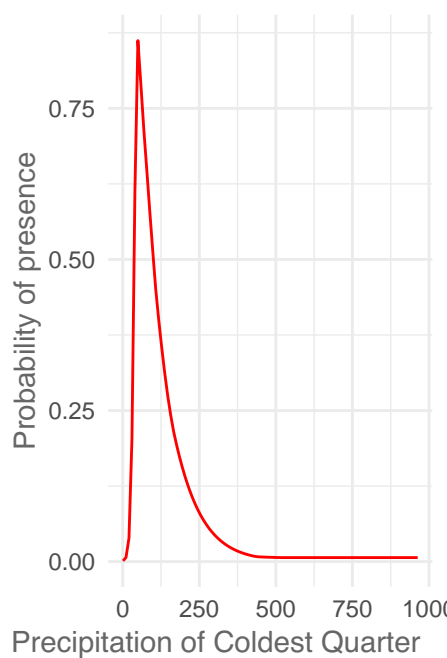

Supplement: Supplemental Information 8 — The variable response of Gopherus agassizii to climatic and environmental predictors. The model identifies influential variables such as temperature and solar radiation, showing how variations in these factors can affect the predicted habitat suitability. [file peerj-13-18568-s008.pdf]

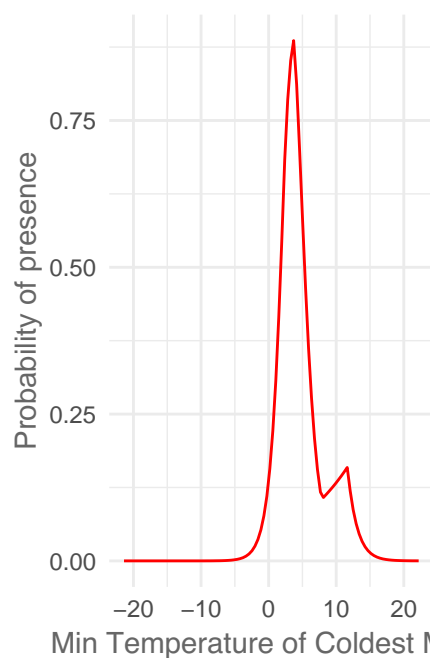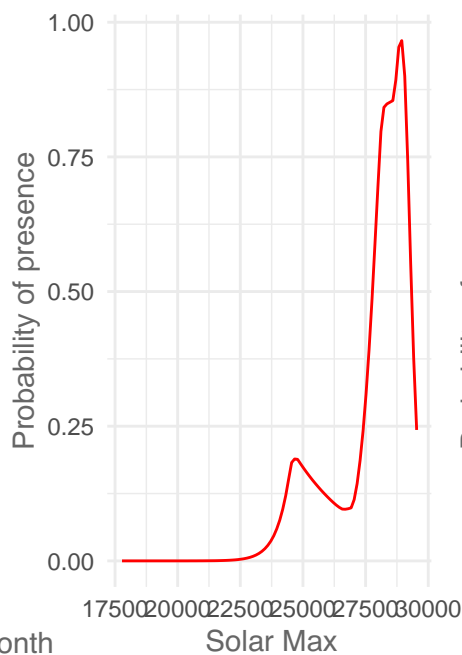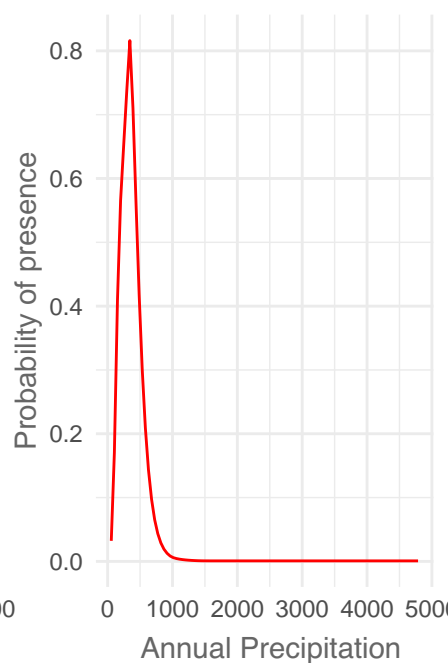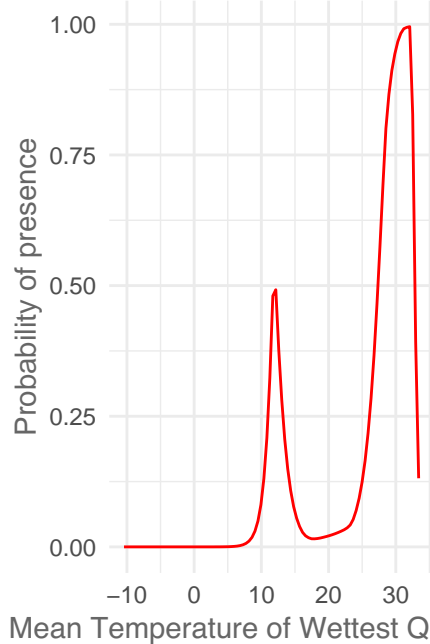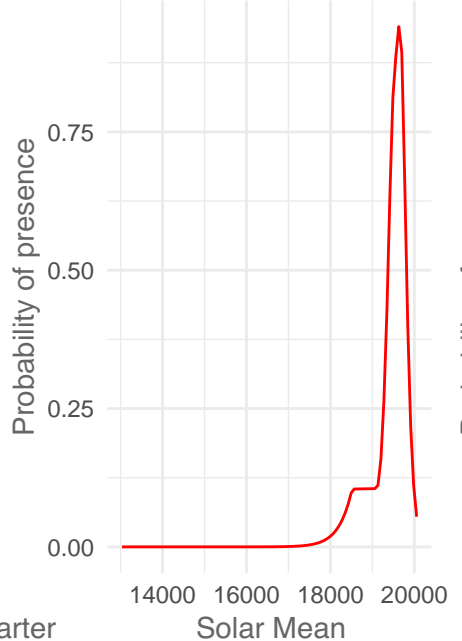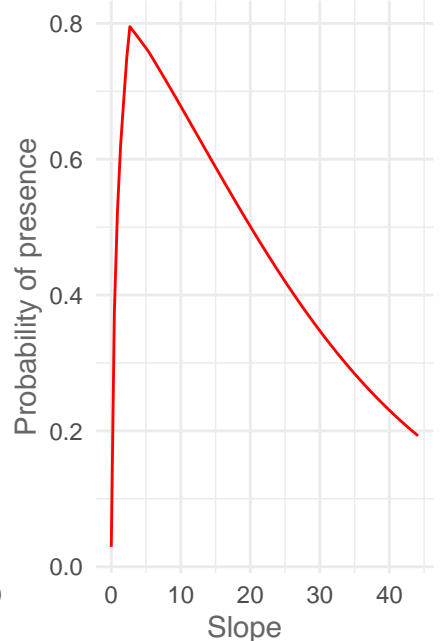

Supplement: Supplemental Information 9 — The variable response model for Gopherus morafkai, showing the influence of key variables, such as temperature and solar radiation, on habitat suitability. The response curves reflect the sensitivity of the species to changes in environmental conditions. [file peerj-13-18568-s009.pdf]

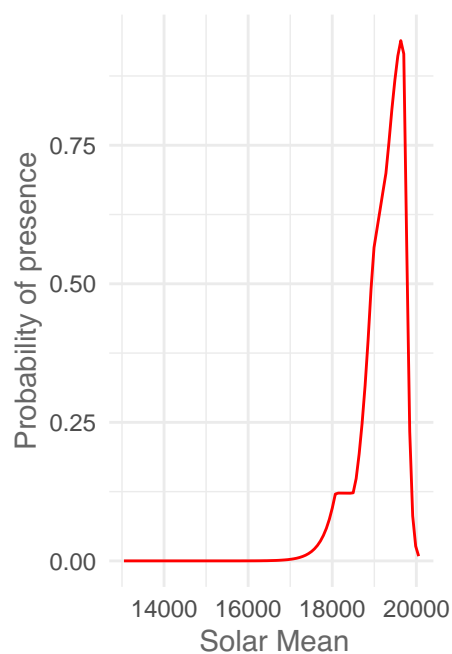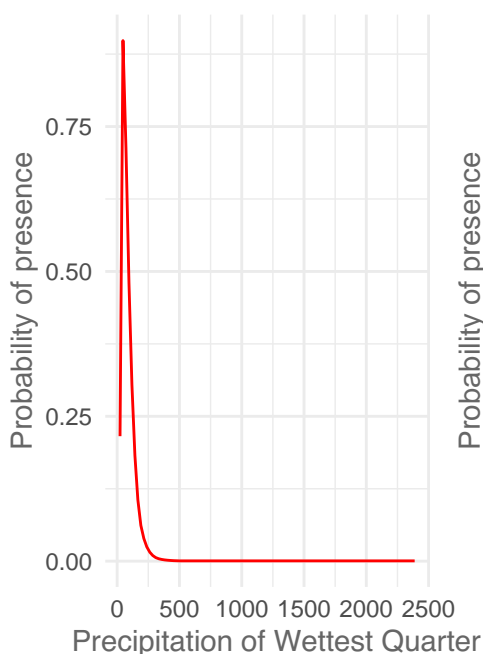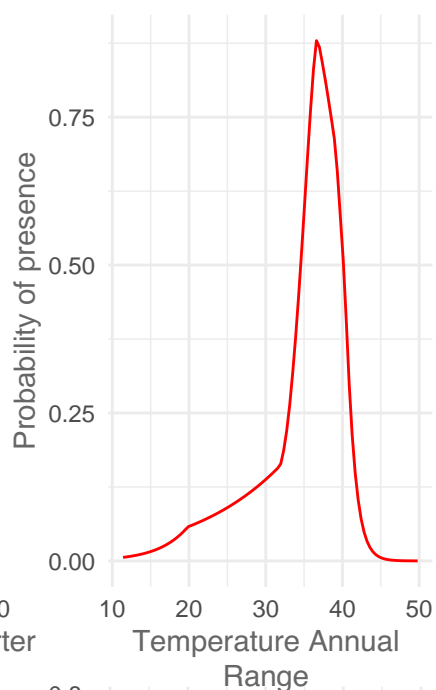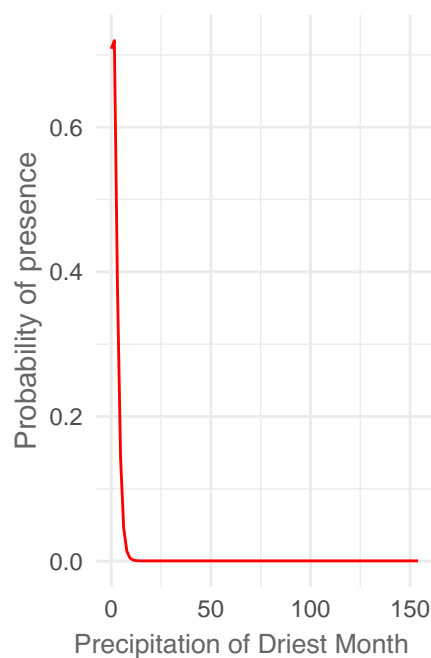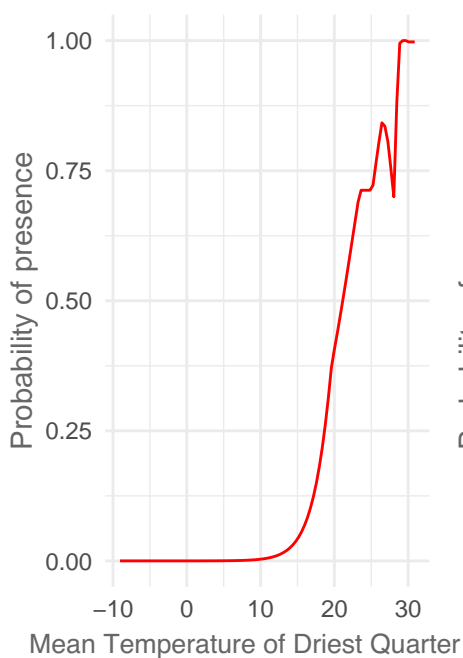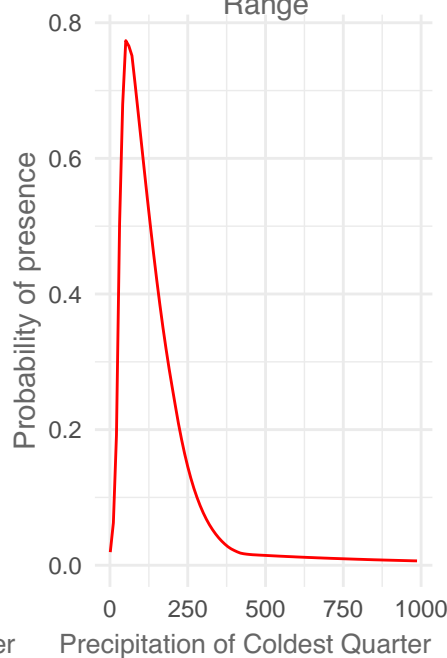

Supplement: Supplemental Information 10 — The variable response model for Toxostoma bendirei , demonstrating how environmental factors, including temperature and solar radiation, influence habitat suitability. [file peerj-13-18568-s010.pdf]

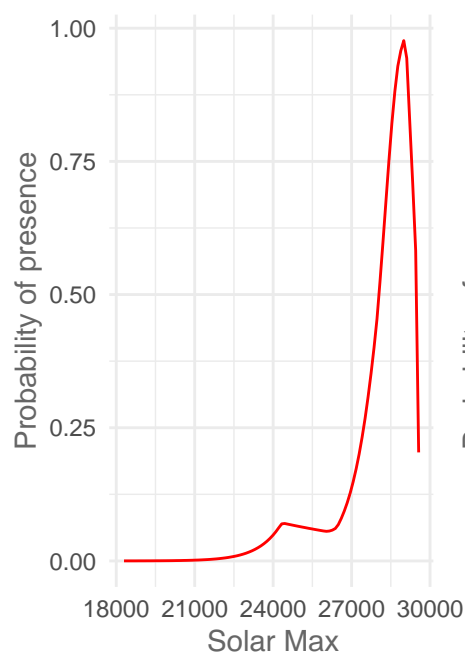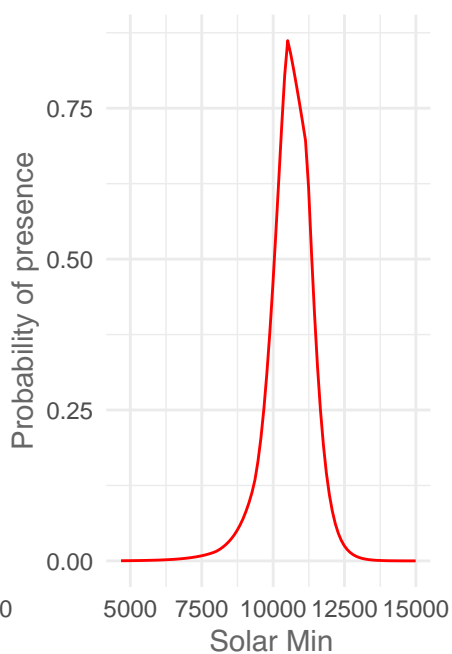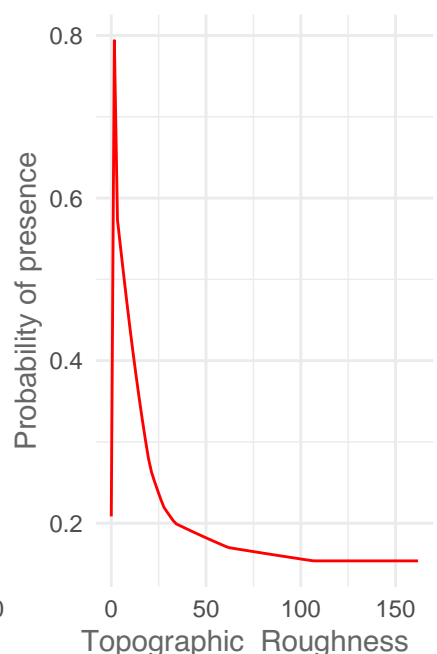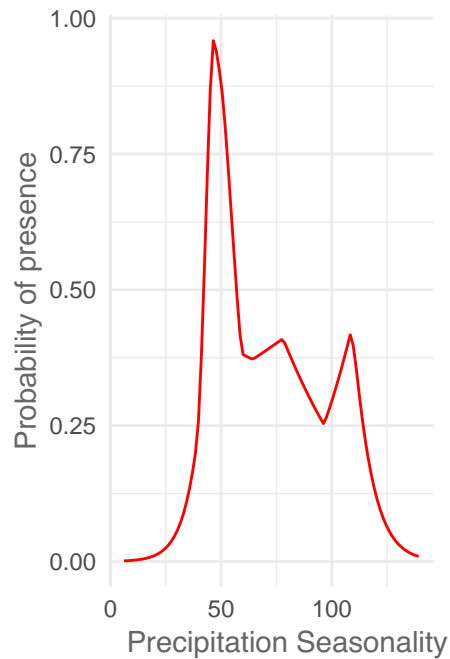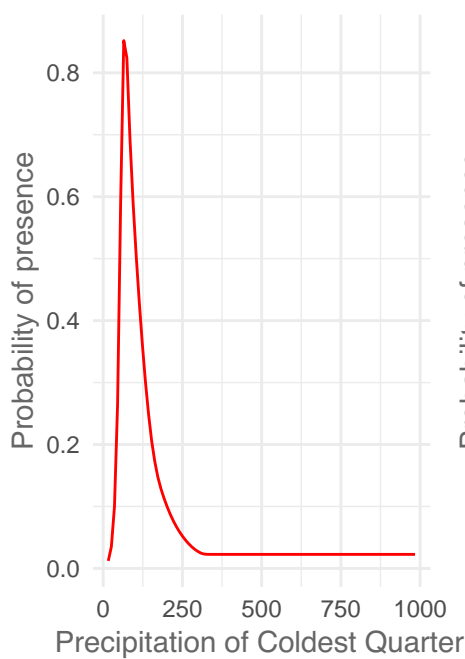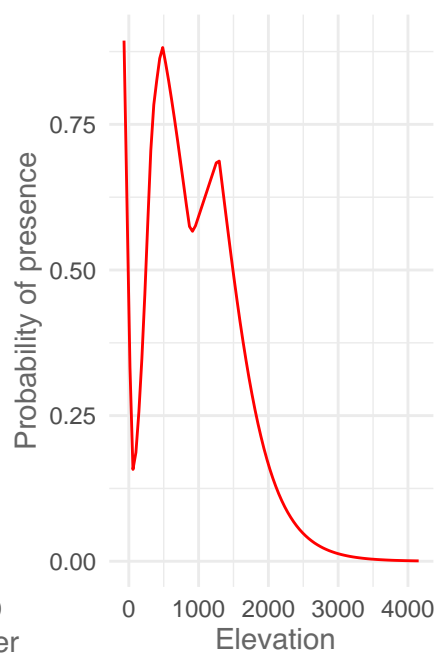

Supplement: Supplemental Information 11 — The variable response model for Toxostoma bendirei, demonstrating how environmental factors, including temperature and solar radiation, influence habitat suitability. [file peerj-13-18568-s011.pdf]
