# Supplementary material for: Mapping species of greatest conservation need and solar energy potential in the arid Southwest for future sustainable development
Source: PeerJ. 2025 Jan 2;13:e18568. doi: 10.7717/peerj.18568 (PMC11700496; doi:10.7717/peerj.18568)
Supplement: Supplemental Information 4 — Orange areas denote regions identified as high priority habitats for the selected species. Yellow regions represent suitable locations for potential SED. [file peerj-13-18568-s004.pdf]

SED Opportunities When Considering Biological Impact

Description -
Includes all target species IUCN extants, also including both resident and breeding extants for Toxostoma Bendirei and Athene Cunicularia.

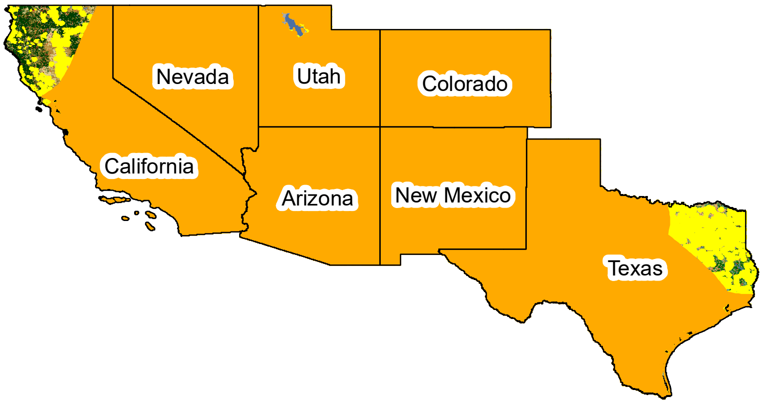

Description -
Includes all target species resident IUCN extants, exclduing breeding extants for Toxostoma Bendirei and Athene Cunicularia.

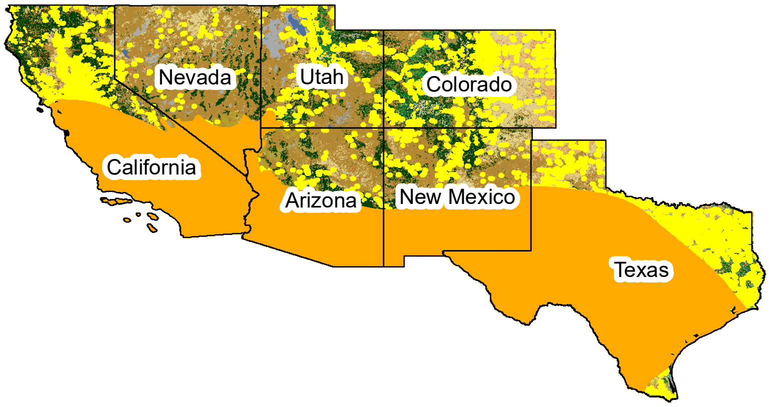

Description -
Includes Toxostoma Locontei, Gopherus Morafkai, Gopherus Agassizii IUCN extants, and resident exant for Toxostoma Bendirei.

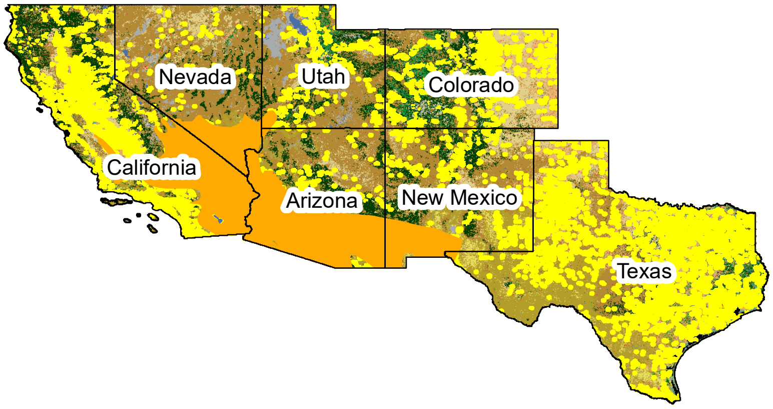

Description -
Includes all nonmigratory IUCN extants, including Toxostoma Lecontei, Gopherus Agassizii, and Gopherus Morafkai.

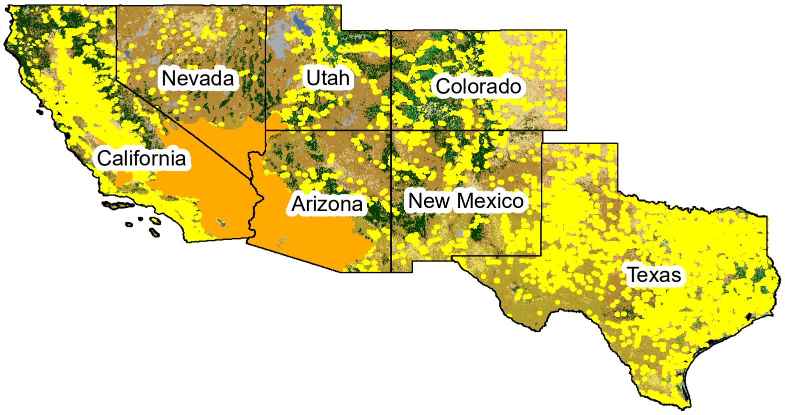

Legend

Target Species High Priority Habitat

Suitable SED Locations

0 250 500 1,000 Miles

N

Author: Kylee Fleckenstein
