# Supplementary material for: Mapping species of greatest conservation need and solar energy potential in the arid Southwest for future sustainable development
Source: PeerJ. 2025 Jan 2;13:e18568. doi: 10.7717/peerj.18568 (PMC11700496; doi:10.7717/peerj.18568)
Supplement: Supplemental Information 5 [file peerj-13-18568-s005.pdf]

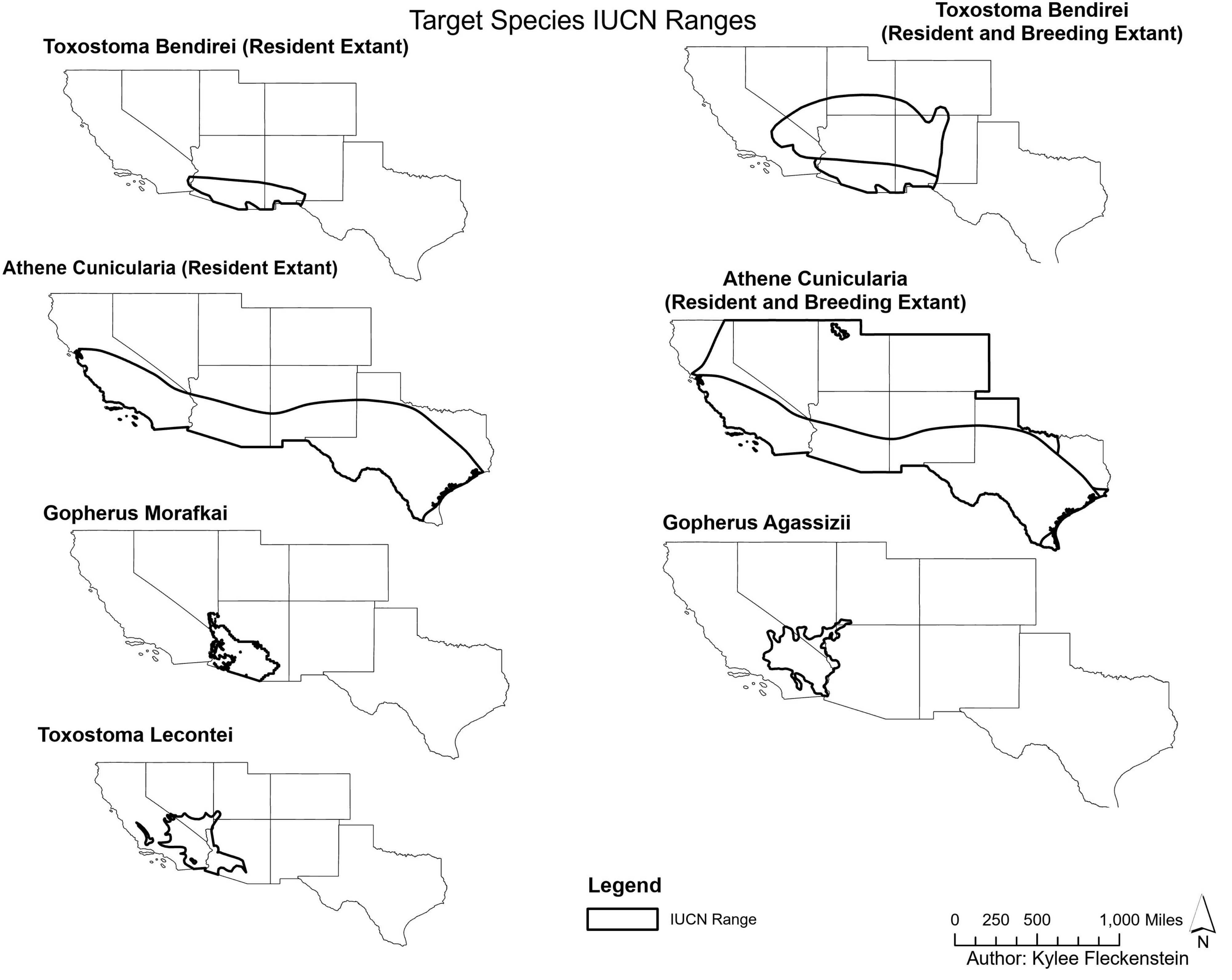
Target Species IUCN Ranges
Toxostoma Bendirei (Resident Extant)
Toxostoma Bendirei
(Resident and Breeding Extant)
Athene Cunicularia (Resident Extant)
Athene Cunicularia
(Resident and Breeding Extant)
Gopherus Morafkai
Gopherus Agassizii
Toxostoma Lecontei
Legend
IUCN Range
0 250 500 1,000 Miles
N
Author: Kylee Fleckenstein
